# Bonding and Strengthening the PLA Biopolymer in Multi-Material Additive Manufacturing

**DOI:** 10.3390/ma15165563

**Published:** 2022-08-13

**Authors:** Emila Brancewicz-Steinmetz, Jacek Sawicki

**Affiliations:** Institute of Materials Science and Engineering, Lodz University of Technology, Stefanowskiego 1/15, 90-924 Lodz, Poland

**Keywords:** multi-material printing, PLA, 3D printing, biopolymers

## Abstract

3D printing is a revolutionary additive manufacturing method that enables rapid prototyping and design flexibility. A variety of thermoplastic polymers can be used in printing. As it is necessary to reduce the consumption of petrochemical resources, alternative solutions are being researched, and the interest in using bioplastics and biocomposites is constantly growing. Often, however, the properties of biopolymers are insufficient and need to be improved to compete with petroleum-based plastics. The paper aims to analyze the available information on elements produced from more than one material, with additive manufacturing resulting from 3D printing using biopolymer Polylactic Acid (PLA). The study notes the possibility of modifying and improving the properties of PLA using layered printing or by modifying PLA filaments. Several modifications improving and changing the properties of PLA were also noted, including printing parameters when combined with other materials: process temperatures, filling, and surface development for various sample geometries.

## 1. Introduction

Polymers are a group of materials used in the automotive [1], medical [2], construction [3], and textile industries [4,5]. The environmental approach to infrastructure requires more and more care for preserving the environment. A significant part of polymers is made from petrochemical products and is not biodegradable [6]. Among the polymers in production, there are biodegradable and non-biodegradable polymers of biological and synthetic origin [7,8,9]. Biodegradable polymers have long been recognized as a possible replacement for materials produced from limited petrochemical resources. Fossil fuels and gases could be minimized and replaced by using polymers from green agricultural resources, reducing global CO_2_ emissions [6].

Polymers have been used in the last five decades: polymer components are manufactured using many technologies such as hot stamping, injection molding, piston injection molding, thermoforming, and many more [10].

3D printing is a revolutionary additive manufacturing technique [11,12,13] that enables rapid prototyping of complex geometric structures [14] and flexibility during designing, even the most demanding structures [15,16,17,18]. Many thermoplastic polymers can be used for 3D printing, including modern biopolymers (i.e., protein- and carbohydrate-based materials) [19].

Applications of additive manufacturing technologies include biomedicine [20,21,22,23,24,25,26,27], dentistry [2], the automotive industry [1,28], aviation [3], and optics [29,30], but also textiles and everyday products [31,32]. The development of printing leads to more and more applications, including conductive, electrically functional, insulating, or semiconductor materials [33]. A wide range of polymers available for 3D printing enables the creation of geometries with shape memory, where structures change their shape under specific external stimuli such as temperature, light, or water [33,34]. When considering additive manufacturing technologies, it is necessary to analyze the types of 3D printing technologies and which of them enable additive manufacturing using polymers. 3D production can be divided into three main categories [35,36]: forming, subtractive, and additive manufacturing.

There are many additive manufacturing technologies using polymers [35]; most of them require the use of pre-prepared polymer material in the form of fiber [37,38], powder [39,40,41,42], or sheets [43]. Other technologies use hardening photosensitive resins [44,45,46,47], where a focused UV laser beam used on the surface of a photopolymer resin hardens molecular chains and solidifies the resin [48,49,50,51].

The constant development of printing technology and rapidly emerging technological innovations make it difficult for young users and industry workers to navigate the polymer printing processes [52] efficiently. The printing process requires a thorough analysis and selection of parameters to predict the properties of objects and obtain the expected results [53,54]. It is necessary to obtain knowledge about the processed materials’ characteristics and analyze the existing information on the chosen applications [55].

As it is necessary to reduce the consumption of petrochemical resources, alternative solutions are being researched, and interest in bioplastics and biocomposites is constantly growing [1,56,57]. Often, however, the properties of biopolymers are insufficient and need to be improved to compete with petroleum-derived plastics. The solution may be using polymer composites [52,58] or combining various materials in 3D printing [59,60,61,62]. The fused deposition modeling (FDM) technology enables fast and cheap manufacturing; moreover, the design of many printers allows for multiple materials—they use two printing nozzles. Two nozzles can work simultaneously with two different materials and make it possible to modify the parameters for each of them. These functions lead to continuous improvement of printing processes [63], and their optimization and flexibility in production.

**Figure 1:**
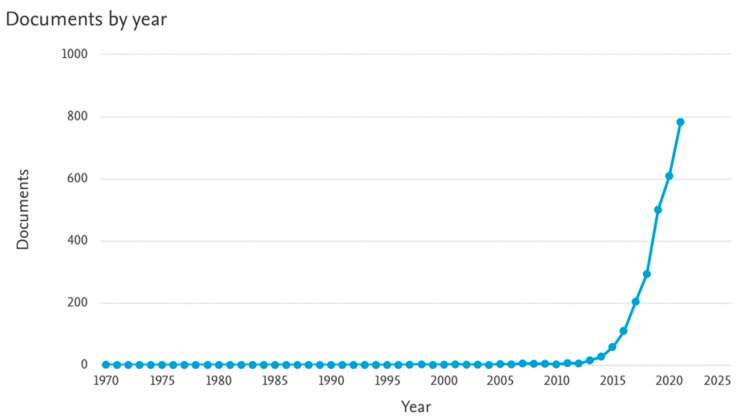
Analyze search results by keywords: ‘PLA printing’ in 2000–2021 [64].

**Figure 2:**
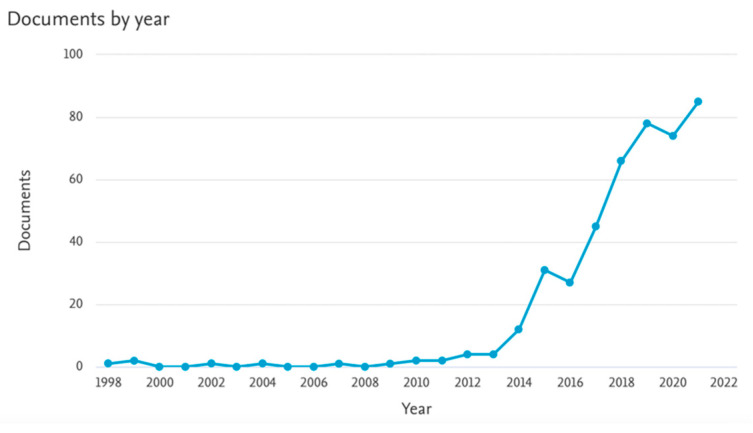
Analyze search results by keywords: ‘multi-material printing’ in 2000–2021 [64].

Significant interest in printing using multiple materials grew from 2013, increasing research papers on this subject. The statistics for 2000–2021 are presented in Figure 1 and Figure 2 [64]. Printing with PLA is being more and more widely researched. In 2021, it appeared in almost 800 papers, while over 80 publications on multi-material printing were published in the same year.

The article is a collection of the latest literature on elements produced from multiple materials with additive manufacturing due to 3D printing using the PLA biopolymer. The mechanical properties of the prints, the quality of bonding, and the possibility of using the products in the industry will be analyzed. The possibilities of modifying and improving PLA properties with layered printing or by modifying PLA filaments will be described.

## 2. Goal of the Review

The selection of papers analyzed in this review was based on specific qualities—the papers should describe using polymer combinations in additive manufacturing processes. Section 3 presents a collection of literature on various manufacturing techniques. This section summarizes publications on additive production with PLA and a set of manufacturing parameters from multiple materials in one process. The recapitulation summarizes the latest knowledge about the constantly tested printing from several materials in one process, including filaments of experimental production.

Section 4 summarizes the results achieved by the researchers and the properties of the objects produced from multiple materials (if any have been tested). Section 5 summarizes, draws conclusions, and outlines research topics that can be conducted after analyzing the entire review.

## 3. 3D Printing with PLA

Multi-material printing using polymers is the subject of research led by many scientists worldwide. The number of materials for printing is practically unlimited [65], especially considering the independent production of filaments or their modification [66,67,68,69], which is becoming easier and is more available for research institutions. One popular and economic [70] printing technique using polymers is the FDM/FFF technology [70,71,72,73,74]. The review is devoted to biodegradable polymers or polymers from biological materials to protect the environment. Common Polylactic Acid (PLA) fibers, combinations with other materials in one-step printing, and modifications and admixtures in commercial filaments were analyzed. The focus was on printing parameters that allow for a reliable process.

### 3.1. Layered Printing with PLA

Polylactic acid (PLA) is a biodegradable material [75] obtained from natural crops by biological fermentation [76]. It is characterized by good stability during printing [77] and a relatively low melting point (180–220 °C), with a glass transition temperature of 60–65 °C [78]. Printing parameters affect the quality of samples and their strength; print orientation, layer thickness, and process temperatures influence the creep strength of polylactide [79].

However, its strength properties may be insufficient for many industrial applications—it needs to be strengthened or modified [80].

To use the ecological potential of biodegradable polymers, material modifications are necessary (e.g., the creation of composites based on PLA). Multi-material 3D printing makes it possible to modify the properties of objects using various polymers [81] and additives in one printing process [82]. Annealing can be used to stereo-complexity 3D samples made of PLA—placing the samples in a vacuum oven at 50 °C for 24 h and then subjecting them to a temperature of 160–210 °C for an hour. Results show [83] that steam treating the printed parts with acetone significantly improves the surface finish of the products, with minimal variations in the geometric accuracy of products after treatment. Manipulation of the printing and heat treatment process parameters may lead to specific properties and structures of objects [76]. Multi-material printing is often performed using two nozzles, where the printer feeds material from separate feeders. One nozzle can also be used, but then the participation of the printer operator who changes the filament during the process is necessary.

In an article written by Ribeiro M. et al. [84], the effect of the bonding surfaces of materials in printing with multiple materials (face-to-face interface) was investigated, and a solution was proposed in the form of mechanical interlocking systems on bonded layers—three different geometries were tested (T-shape, U-shape, and dovetail shape). The positive influence of the mechanical bonding of TPU and PLA on the elasticity of the samples was demonstrated. The overlapping of material boundaries contributed to the achievement of higher Young’s modulus values.

The research [77] analyzed the influence of surface development and roughness on the interlayer adhesion of PLA and TPU (thermoplastic polyurethane). The best combinations of surface pattern bonds common for both printing orders with polymers in cylindrical samples were indicated: pattern concentric for the combination of PLA/TPU (shear strength 0.43 MPa) and TPU/PLA (shear strength 0.38 MPa). At the same time, the best surface development for the TPU/PLA sequence is the TPU-linear pattern at 0° and the PLA-linear pattern at 45° (shear strength 0.63 MPa).

To explore the topic of layered bonds of materials during printing, it is worth analyzing the work of Tamburrino et al. [85], where the following aspects were investigated: the order of printed materials, the pattern development of the upper and lower printed layers and their influence on the interlayer adhesion strength. Three pairs of PLA-TPU, CPE-TPU, and CPE-PLA materials were tested. The use of a lower filling density (80%), compared to 100% density, harmed the adhesive strength; at the same time, a solution was proposed in the form of a Mechanical Interlocking mechanism (two materials were combined on several central print layers, a frame made of one material and an internal space was filled with the second material so that the materials overlapped on several print layers), which increased the adhesive strength of the samples (for the PLA-TPU connection, thanks to the mechanism, an increase in Peak stress from 0.28 MPa to 1.32 MPa was observed).

Research led by Kumar S. et al. [86] concerns multi-material printing based on Polylactic Acid (PLA). Several mixtures were combined: pure PLA, PLA with PVC admixture, PLA with wood powder, and PLA with magnetite (Fe_3_O_4_). During the experiments, the parameters of the filling—at which the highest breaking strength of 41.65 MPa was achieved—were determined. The best filling parameters were: infill density of 100%, infill angle of 45°, and infill speed of 90 mm/s. At the same time, the research showed a negative impact of lower print densities and their roughness on the quality, strength, and mechanical properties of objects compared to samples printed with a high density of up to 100%.

Multi-material prints made of ABS and PLA reinforced with carbon fiber were tested to determine the influence of printing parameters on the strength of interfacial bonding [87]. The optimum printing parameters were determined (a printing speed of 50.54 mm/s, infill density of 79.82%, layer height of 0.15, and a layer thickness ratio of 0.49). It should be noted that PLA is less toxic than ABS. Attempts to use PLA reduce environmental pollution with volatile organic compounds such as, for example, styrene, butanol, cyclohexanone, and ethylbenzene [88]. From the point of view of the respiratory health of printer users, the preferred combinations of polymers in printing are PLA, PET, and TPU, as those materials used at lower temperatures have lower FP (fine particle emissions) than ABS. Moreover, when printing with ABS, an increase in inhaled nitric oxide (FeNO-nitric oxide) and the presence of an unpleasant odor are observed compared to printing with PLA [89].

During the impact tests of mesh samples printed with the FDM technology, it was found that the use of external walls in such structures strengthens the impact strength of the samples by 60%. The combination of ABS and C-PLA (carbon fiber + polylactic acid) material shows a much higher impact strength (280 to 365%) compared to samples from C-PLA. The impact strength of the samples in this study was from 7672.9 to 23,465.6 kJ/m^2^ [90].

Laminar composites are widely used in the industry. To manufacture them in 3D printing processes, manufacturing parameters should be selected to strengthen the objects. Such parameters include low printing speed, layer height, and the clad ratio [91]. Strength tests were carried out on samples made of two materials—ABS and PLA reinforced with carbon fiber. It has been shown that multi-material samples are characterized by higher strength than individual materials. The following parameters were considered the best: speed 1⁄4 20 mm/s, infill density 1⁄4 67.838%, layer height 1⁄4 0.23 mm, and clad ratio 1⁄4 0.25. The highest values on strength tests were elastic modulus = 2204.45 MPa, ultimate strength = 51.34 MPa and elongation = 9%.

3D printing from multiple recycled polymers such as ABS, PLA, and HIPS is feasible because these thermoplastics have a similar heat input during heating (13.63 mJ for ABS, 14.71 mJ for PLA, and 11.71 mJ for HIPS). Compared to single-material 3D printing, multi-material 3D printing offers more flexibility to functional prototypes (with completely different/improved multi-dimensional properties) [92]. Considering sandwich structures made of several materials, samples with HIPS outer layers and a rectilinear ABS core showed the worst performance, with average tensile stress of 22.21 MPa and Young’s modulus of 992.02 MPa—which is less than 50% and 28%, respectively. Of the best layer structure tested, it was PLA-ABS-PLA. The average values were 44.40 MPa for tensile strength and 1364.25 MPa for Young’s modulus for the best configuration. Moreover, the elongation at break (6.14 mm) for this configuration was higher than the homogeneous material [93]. The PLA/ABS/PLA layered structure showed higher tensile strength than the pure ABS sample, which leads to the conclusion that the strengthening of ABS with PLA biopolymer is effective for selected applications.

The quality of the interlayer bond is influenced by the surface development and the surface finish pattern. At the same time, the printing parameters have a very significant impact on wettability, and attention should be paid to the optimization of process parameters combination selection: layer thickness, filling method, and printing speed [94]. In an experiment, a combination of process parameters such as the mesh fill method and a layer thickness of 0.25 mm can be used to produce parts with the maximum bond angle. To modify the surface morphology of multi-material prints, ICP-CFx (inductively coupled plasma and coated by fluorocarbon-based material) treatment is used. The treated PLA/PE-HD (high-density polyethylene) surface in the proportion 90/10 showed a bond angle of 121.6°, 36° higher than the bond angle measured on the untreated surface [95].

The quality of material bonds is directly related to sample type. During their research, Lopes et al. proved the negative influence of geometric boundaries between the same printed material from two embossing heads; the lack of chemical affinity between the materials worsens the effect—a decrease in Young’s modulus tensile strength is observed [96].

Kumar S. et al. [97], on multi-material printing in FDM technology, studied the combination of PLA with a polyamide-titanium dioxide (PA6-TiO_2_) composite. The best printing parameters, such as a printing speed of 90 mm/s and a rectilinear filling pattern, were determined. The influence of the number of layers of each material on sample peak strength was also investigated—it was found that 5 PLA layers and five composite layers were the best combinations. The selected combination showed higher strength (61 MPa) than that of pure PLA (42–45 MPa)—the same was discovered for thermal stability. Pin on disc wear tests showed that PA6/TiO_2_ material consumes less material compared to PLA; hence, samples based on PA6/TiO_2_ can be used for applications with high wear rates [98].

Using PLA and poly (3-hydroxybutyrate) PHB, thermal stability and interfacial adhesion of prints can be improved using cellulose nanocrystals and DCP dicumyl peroxide as a crosslinking agent [99]. A nanomaterial that meets the high standards of engineering applications was produced by obtaining cellulose from plum pits. PLA/PBAT/PBS composite (polylactic acid/poly (butylene adipate-co-terephthalate)/poly (butylene succinate) with nano talc was discovered to be the best combination and the best roughness and dimensional accuracy parameters were obtained for the proportion of 70/10/20/10 [100]. A summary of information on layered printing is presented in Table 1.

### 3.2. Modifications of the PLA Filament

A constantly developing trend in 3D printing is the modification of ready-made polymer filaments, enriching them with biodegradable components and testing their properties. An example of this approach is the research of Singh M. et al. [101], where shear resistance using cancellous screws of objects printed in FDM technology with PLA filament with the addition of almond skin powder was examined. The study suggested that the maximum peak shear strength (23.02 MPa) and the maximum shear strength at break (22.90 MPa) were observed for the honeycomb fill pattern at 100% screw insertion and a 30° rake angle.

An innovative approach consisting of overwhelmed physical interlocking and minimum chemical grafting in the production of PLA filament with polypropylene for 3D printing ensured high structural stability (mechanical and intermolecular) concerning thermal degradation (compared with pure PLA) [102].

Attempts to strengthen PLA with silicon nanocomposites (clay nanocomposite) [103] proved that the addition of nanoclay increased thermal stability and the modulus of elasticity. The samples were made of pure PLA and PLA with nanoclay. The color of samples with nano clay changed. As the printing temperature increased, the samples turned brown, but it is worth noting that at the same time, despite the color change, they became more transparent. This indicates another essential aspect when printing from composites, where the filament composition and the printing parameters are crucial for the design and further applications of objects.

Enrichment of PLA with silica (silica-silicon dioxide SiO_2_) strengthens PLA: adding 10% silica by weight increased tensile strength from 62.8 MPa for pure PLA to 121 MPa for enriched PLA [104]. As a natural material, silica can strengthen polymers and create biodegradable composites for 3D printing. The silica additives can enhance the handling and quality performance of composites and thermoplastic polymers because of their diverse potential.

Attempts to strengthen PLA with flax fibers indicated the need to plan the additive manufacturing process carefully. The researchers indicated [105] that flax fibers could strengthen the samples, but their disadvantages, such as intra-filament porosity and the surface condition, should be eliminated. These disadvantages contributed to internal material gaps and the weakening of bonds. The fatigue behavior of specimens made of PLA and PLA reinforced with filler based on pinewood, bamboo, and cork using FDM was tested [106]. Testing did not significantly affect the change in tensile strength and associated durability during this loading interval for PLA-based materials reinforced with natural filler.

SEM analysis showed the presence of porosity, interlayer disturbances, and at the same time, good interfacial compatibility of PLA with the natural filler. Under cyclic loading, the visco-elastic behavior of the tested materials was found to increase with increasing values of cyclic loading of 30%, 50%, and 70%, and the permanent deformation of the tested materials, i.e., viscoelastic behavior (creep), also increased.

In Guessasma S. et al. [107], wood-based fibers’ microstructure and mechanical properties were investigated in FDM technology (with experimental and numerical methods) about the PLA/PHA wood printing temperature. The optimum printing temperature was determined—220 °C for printing with wood-based filaments while maintaining adequate tensile strength, compared to objects printed at temperatures in a range of 210–250 °C. Water adsorption and desorption properties of wood change the dimensions of wooden objects, which is often considered a disadvantage. Using those properties in filament modification can lead to producing objects that change shape with humidity. PLA was modified by adding different wood contents to produce shape-changing double-layer actuators. The higher the wood content, the greater the observed shape change. PLA with wood can be used in 3D printing elements induced by humidity control—changing shape under changing climatic conditions [108].

Polymers for 3D printing can be given bioactive properties directly from natural extracts (e.g., from Mango extracts), which will allow the use of 3D printing with polymers in medicine [109]. Polymers could be used as carriers for medicinal substances released only after implantation in the patient’s body. The use of polylactic acid with methotrexate or an anti-cancer drug (PLA/MTX) made it possible to print a frame that releases the active ingredient at the implantation site for more than 30 days, reducing side effects caused by injection or oral administration. This makes it possible to 3D print frames and use them in drug delivery [110]. Filament made of biomaterials such as PLA, polycaprolactone PCL, and hydroxyapatite HA became the building blocks of interlocking nails used for bone fractures in dogs. The highest compressive strengths of 82.72 MPa and tensile strengths of 52.05 MPa were achieved with the highest tested hydroxyapatite content of 15% [111]. Assessment of the cytotoxicity of the PLA/PCL/HA combination showed that the cells could be viable and increase in the frames. The most favorable PLA/PCL weight ratio in biocompatibility, viability, and osseointegration was 70/30 [111]. A strong interaction between PLA and HA resulted in the high mechanical strength of the composite [112]. After mechanical testing, the optimum ratio for biological research and 3D printing was selected. Biological experiments showed that the synthesized PLA/HA composite had excellent in-vitro viability. HA/PLA (10:90) had the highest mechanical strength comparable to natural bone among the various tested HA to PLA ratios. At the same time, the HA/PLA sample (10:90) showed excellent printability in 3D bioprinting using the FDM approach [110]. Biopolymer-based materials have the potential for use in prosthetic components, e.g., acetabular components in total hip prosthesis [113,114].

The potential application of the TPU and PLA polymer composition is the production of antibacterial wound dressings using 3D printing. The mechanical, structural, and microscopic analysis and degradation allowed the selection of the most promising combination for further antibacterial modification (filament COMP-7,5PLA: consists of 12 parts of TPU filament and 1 part of PLA). It has been proven that the antibiotic amikacin is stable during extrusion at elevated temperatures, which, in combination with biodegradable PLA, makes it possible to produce short-term implants [115].

3D printing is used for the production of personal protective equipment. TPU and PLA polymers allow re-sterilization; therefore, their use would reduce the amount of biomedical waste [116] due to the possibility of multiple sterilizations and reusing the same elements.

In deliberations [117] on the environmentally friendly, rapidly degradable plastic-enzyme composites, Polycaprolactone/Amano lipase (PCL/AL), PLA, was used as the basis for the application of composites on complex structures.

Blending thermoplastic polyurethane (TPU) with polylactic acid (PLA) is a proven method of obtaining a mechanically more robust material. The addition of graphene oxide (GO) is increasingly used in polymer nanocomposites to customize their properties further. The addition of GO significantly improved the mechanical properties of the polymer matrix; 167% in terms of the compression modulus and 75.5% for the tensile modulus [118]. Cell viability, bonding, proliferation, and differentiation assays using MG-63 osteosarcoma cells have shown that PLA/GO frames are biocompatible and promote cell proliferation and mineralization more effectively than pure PLA frames [119]. The 3D-printed nanocomposite is a promising frame with the appropriate mechanical properties and cytocompatibility that could enable osseointegration and bone formation. A model of the trachea for tissue engineering was developed [120], consisting of multilevel structural polylactic acid (PLA) membranes surrounding thermoplastic polyurethane (TPU) skeletons—polymers were modified with GO-IL (ionic liquid) graphene oxide. The in-vivo result confirmed that the subjects displayed favorable biocompatibility and promoted tissue regeneration.

Creating multi-material objects in FDM technology can be enriched by friction welding; however, it is necessary to achieve consistent rheological properties (e.g., by reinforcing the filaments with aluminum powder) [121]. The addition of aluminum to polymers makes it possible to increase the melting point and, therefore, allows them to be used in joints where the difference in melting points of the two materials is so significant that, due to dissimilarities in their melt flow properties, welding is impossible. Information on eligible filaments is summarized in Table 2.

## 4. Electrical Conductivity Applications

Polylactic acid (PLA) is the most widely used polymer in many areas since it is biodegradable, environmentally friendly, and biocompatible [122].

Modifications of PLA composites with proper additives are the most useful technique for improving the properties of the 3D-printed parts obtained by the FDM method [123]. This article analyzed the strength properties obtained for prints from PLA and modified/strengthened PLA. Deliberations on industry demand need to be raised, as does the possibility of using polylactide as a base for elements with electrical, electromagnetic, and conductive properties.

Researchers presented the electromagnetic interference properties of multi-walled carbon nanotubes (MWCNTs) as a filler in PLA/PEG polymer matrix. The results showed that the dielectric properties increased with increased MWCNTs filler [124].

The ability to adjust shielding properties through the fabrication of polymer composites with pores was tested by using graphene nanoplatelet/poly-lactic acid materials. Researchers fabricated non-perfect electrical conductor-backed porous composites, and explored the effect of filler aspect ratio and pore geometry on electromagnetic interference shielding properties. Composite structures demonstrated decreased fractions of reflected power and increased fractions of absorbed power over most of the X Band because of the addition of periodically arranged cylindrical pores [125].

The electromagnetic and thermal characteristics exhibited by the nanocomposites make them suitable for packaging applications of electronic devices with electromagnetic interference shielding and thermal dissipation features. The research showed [126] that the combination of notable electrical, thermal, and electromagnetic properties of the carbon fillers, in concentrations above the percolation threshold, together with the good processability of the PLA matrix gives rise to innovative filaments for 3D printing.

The analysts [127] obtained results that can maintain in interpreting the influence of processing on the properties of the final products based on PLA composites. The crystallinity of the 3D printed samples is more highly matched to the filament and hot-pressed samples, but this structural feature has a slight effect on the electrical and tensile properties. The type of structural organization of multiwall carbon nanotubes, graphene nanoplatelets, and combined fillers in the matrix polymer is found to be determinant for the electrical and tensile properties.

In research [128], the effect of graphene/spherical graphite ratio on the microwave absorbing properties and mechanical properties of PLA/TPU composites was specifically tested. It was found that when the ratio of graphene/spherical graphite was small (0:5, 1:4), the dielectric loss and attenuation ability of the composites were stronger, and the impedance matched better. The graphene/spherical graphite ratio was large (5:0, 4:1), and the composites had high strength and toughness. When the ratio of graphene/spherical graphite was moderate (2:3, 3:2), it could retain the absorbing and mechanical properties of the absorbing materials. In work [129], the measurements showed a significant increase in the dielectric values, with the addition of polylactic acid nanocomposite with graphene.

The design of two mushroom metasurfaces using 3D printing with conductive PLA filaments for the metallic parts was presented in the research [130]. Measurement results of the 3D-printed metasurfaces show the appearance of the stopbands at the simulated expected frequencies. It is difficult to find filaments on the market that are compatible with low-cost 3D printing and have conductive properties suitable for even the most demanding applications. Preparation of PLA composites with proper additives is the most useful technique for improving the properties of the 3D-printed PLA parts purchased by the FDM method. Smart 3D structures with embedded and printed sensory elements (the sensor was based on the conductive PLA) were examined in the research [131]. The researchers were focused on dynamic measurements of the strain and considered the theoretical background of the piezoresistivity of conductive PLA materials. The capability of FDM 3D-printed sensors to perform dynamic strain measurements was proven up to 800 Hz. Results support future applications of smart systems with embedded sensory elements.

Scientist designed electrodes efficiently for electrochemical sensing in the food industry. The results showed a basis for the promising application of detecting and quantifying 3-monochloropropane-1,2-diol (food contaminant known for its potential of being carcinogenic).

The nanocomposite [132] of reduced graphene/PLA/PEG matrix was prepared via the melt blending method. Researchers presented materials that may be used as radiation absorbers if their reflection could be reduced via impedance matching at the surface, as in structure-engineered shieldings, such as multilayer structures, or via foaming.

To improve the electrical properties and maintain sufficient strength for 3D printing, nano carbon was infused in PLA. Researchers [133] provide a perspective use of this filament for fabrication of electrical wires in 3D printed robots, drones, or prosthetics.

Researchers showed [134] the development of 3D printed, highly stretchable and sensitive strain sensors using graphene-based composites. The printing of graphene/PLA/TPU in a meander sign wavelike structure significantly enhanced the stretchability by up to 4 folds, to over 30% strain, while supporting the sensitivity of common graphene-based strain sensors.

Manufacturing conductive filaments make it possible to widely use 3D prints for structural and carrier applications and electrical applications. Graphene, in combination with polylactide, can be used to produce heating elements (e.g., for textile applications and the production of heated clothing). Low surface resistance was demonstrated at high surface temperature (83.6 °C) for 0° printing [135]. When using commercial graphene-enriched fibers in laboratory conditions, contamination should be considered (e.g., in the form of metals), which may affect the electrochemical properties of prints [136].

A low-cost composite material was presented in the research [137] suitable for the production of a multifunctional filament with improved electrical and thermal properties for different fused deposition modeling composites were obtained. A synergistic effect was observed in the PLA with graphene nanoplates and multi-walled carbon nanotube hybrid composites when combining graphene nanoplates and carbon nanotubes at a ratio of 3% GNP/3% CNT and 1.5% GNP: 4.5% CNT, showing higher electrical conductivity concerning the systems incorporating individual CNTs and GNPs at the same overall filler concentration.

Researchers proposed [138] a filament indicated for the FDM process with improved dielectric and thermal properties, compared to the pure PLA. Relative permittivity reaches the value of 5.35 × 10^3^ much greater for 12 wt% of multi-walled carbon nanotubes than that of 3.7 measured for unfilled PLA. The thermal conductivity of the enhancement with 12 wt% of graphene nanoplatelets is about 261% concerning the thermal behavior of the neat polymer. In this research, innovative material that appears promising for the electromagnetic field and heat transfer was proposed. The thermal conductivity of the PLA loaded with 12 wt% graphene nanoplatelets is 263% higher than that of pure polymer, whereas an improvement of about 99% and 190% is detected for the PLA matrix loaded with multi-walled carbon nanotubes and both fillers, respectively [126].

Many parameters of fused deposition modeling (FDM) influence the resulting mechanical properties. This can become a key aspect if parts are intended for commercial applications. When printing from multi-material filaments, in order to achieve suitable quality, the printing parameters should be analyzed—the research [139] on the optimization of these parameters is a guidance for users, for example, a study on the influence of nozzle temperature and infill line orientations (PLA+ carbon fiber). Scientists attained maximum tensile properties for temperature 230 °C and printing orientations [0°, 15°, −15°].

In experiments [140] of quasi-static tensile tests when printing from PLA–graphene filament, researchers obtained that fatigue lifetime clearly depends on the process parameters as well as the loading amplitude and frequency (when the frequency is 80 Hz, the coupling effect of thermal and mechanical fatigue causes self-heating, which decreases the fatigue lifetime). Preparation of PLA composites with suitable additives is the most useful technique to improve the properties of the 3D-printed PLA parts obtained by the FDM method.

## 5. Multi-Material Printing-FDM Capabilities

Multi-material printing is an often-analyzed method of additive manufacturing. 3D printing technology FDM deposits filaments layer by layer, and some advanced geometries require using support material [141,142], as the selection of process variables plays a crucial role to measure the quality of FDM processed components. Having a multi-material 3D printer allows you to print multiple filaments (one support filament, like soluble material PVA, and the filament for the final part). This helps to ensure producers achieve their perfect design, no matter how complex [143].

A common operation during printing is the use of multi-colored materials or several spools of the same polymer, but of a different color. Using this procedure allows for obtaining visually attractive prints. Multi-material printing differs from multicolor printing and uses two different polymers to make one part in a single work step [144]. This combines the advantages of two or more different materials in one component [145].

When printing from multiple materials with different properties and melting points, it is possible to find tips on how to ensure error-free application in online forums. Generally, methods can be split into two groups: single hot end methods and multiple hot end methods. Multi-material printing on a printer with one nozzle can be carried out by:

- manual change of material between layers [146] (it is necessary to pause the printing, change the spool, clean the nozzle, usually manually change the temperature, resume printing, and change the spool each time when printing with a different material)
- the use of accessory equipment that combine several filaments at a given stage of printing and enable printing from several polymers using one combined filament fiber.

There are various additives available on the market that enable multi-color and multi-material printing [147,148,149]: e.g., 3Dfeedy, 3D Chameleon, Mosaic Manufacturing Palette.

Such equipment works by precisely cutting and splicing various materials and feeding the printer a single, continuous length of filament, which can be printed in much the same way as printing with a single material. The devices have programmed parameters for connecting different polymers—their combinations are constantly updated and improved. Each material combination (e.g., PLA-PLA, PETG-PETG, PLA-TPU) has a variety of different settings that can be manipulated to increase the quality and reliability of splices.

Multiple hot end methods are suitable for working with multiple materials. Printers with two nozzles are to enable work when creating objects from the base and support material (e.g., water-soluble) [143], but they can also be used for printing from several building polymers. Each nozzle in the printer can be programmed separately (it is possible to set separate printing parameters, e.g., speed and temperature). The accessories described above can be used for each of the nozzles, as their use increases the possibilities of combinations and the number of materials combined in one printout.

When printing from different polymers, it is often suggested to combine them by analogy to the properties; similar filaments (materials with the same base polymer or similar processing temperatures) can be firmly bonded. This no longer functions when the difference between processing temperatures is too great: one of the two materials may not remain stable during processing or may even be subjected to damaging temperatures. To be certain of the connection, there is an interlocking design in which the two phases are combined [145].

This article presents the researchers’ position on multi-material printing and filament modification. The described and summarized studies present the tested printing parameters for the strength and quality of combinations of various materials. When planning multi-material printing, a process plan adjusted to the hardware capabilities should be established. Based on the strength tests of samples and guides from other printer users, we adjust the printing parameters to the filaments from which the item will be printed.

## 6. Plastic Waste and Economics of Additive Manufacturing

The economics of additive manufacturing relates to the cost of manufacturing, the value of finished goods, and their utility [150]. According to a report (Wohlers Report) from 2022 [151], the Additive Manufacturing industry grew by 19.5% in 2021. The pandemic significantly impacted the growth from the 7.5% recorded in 2020. Growth and investment in additive manufacturing are targeted at, among others, the healthcare, aerospace, automotive, and energy industries. The report notes the increased use of AM technology for serial production, as evidenced by the increase in the consumption of polymer powder (an increase of 43.3%). Polymer powders overtook photopolymers and became the most used materials in AM. Within a few years, the consumption of polymer powders had increased from 190 million dollars (U.S. dollars) in 2015 to 900 million dollars in 2021. The Covid-19 pandemic has proved that 3D printing can help the supply chain. When demand for protective equipment grew exponentially, the fastest way to obtain it was to use additive technologies and produce universal filaments available on-site [152]. This contributed to meeting the demand by increasing the supply quickly.

3D printing reduces waste and ensures an effective buy-to-fly ratio, which revolutionizes logistics, reducing costs and time of goods distribution [153]. The environmental approach to 3D printing prompts researchers to use environmentally friendly alternatives and modify polymer matrices [154].

Plastic waste from 3D printing can be reused [155] or degraded. The main recycling methods are biodegradation, recycling using a catalyst or solvent, and the processing and reuse of material.

**Figure 3:**
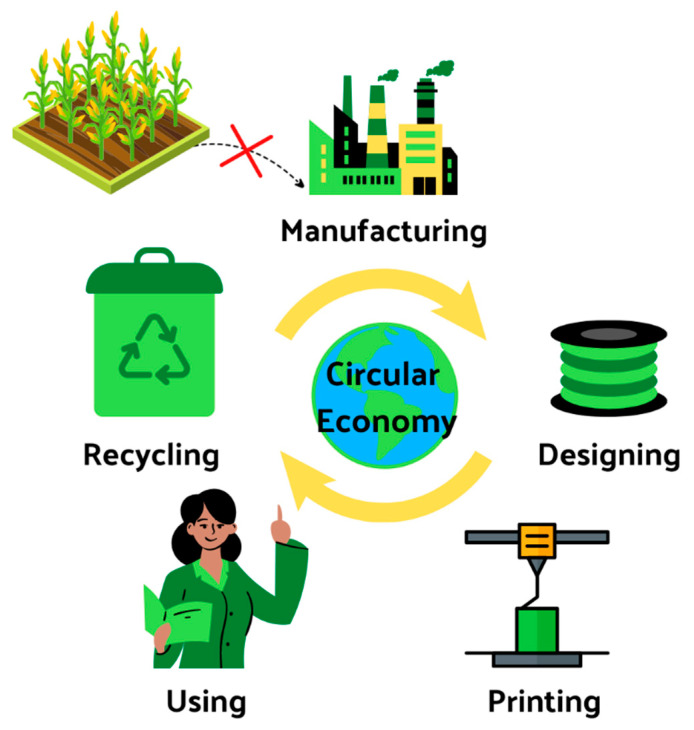
The life cycle of the raw material in a closed cycle—it is necessary to eliminate the need for new resources [158].

Researchers [156] characterized the PLA wastes generated in 3D printing processes and evaluated the effectiveness of their heterogeneity on the technical feasibility of mechanical recycling (two PLA 3D printing wastes were used: waste coming from a well-known PLA grade, and a mixture of PLA 3D printing residues). Recycled material obtained from the waste of a well-known PLA shows good properties, like those for non-used material. However, the recycled material obtained from mixed PLA waste shows lower viscosity values, higher crystallization ability, and less transparency. Results highlight that special attention should be paid to the sorting and characterization of the 3D waste. A circular economy can lead to economic growth where, in the name of the ‘reduce, reuse, and resource’ [157] principle, the consumption of raw materials, energy, and—above all—emissions will be limited. The use of local waste and its processing in local production translates into efficiency and effectiveness in the circulation of materials [158]. Figure 3 shows the cycle of raw material circulation. Initially, it is necessary to obtain materials such as PLA from corn, but after satisfying the market needs, elimination of this stage will lead to creating a circular economy model.

## 7. Discussion and Conclusions

The review determined that:

- PLA is a biopolymer that, compared to other polymers used for 3D printing, emits fewer harmful particles into the atmosphere during extrusion, and thus pollutes the environment less and reduces the risk of respiratory diseases for printer operators.
- the ability to perform multiple sterilizations of ABS and PLA combinations makes their use possible in the medical industry. It may lead to waste reduction due to the possibility of using the products multiple times.
- the authors are researching various geometries of samples, making it possible for users to select the tests closest to the geometry of specific commercial elements.
- annealing or friction welding of printed objects can lead to improvement of their strength properties.
- to strengthen the bonds, dicumylt peroxide (DCP) can be used as a crosslinking agent.
- the use of locking mechanisms in the form of overlapping filaments improves the quality of the samples and the strength of the bonds between the materials.
- for selected material bonds, the negative influence of the lower filling on the strength of the samples was indicated compared to 100% filling.
- parameters influencing the quality of printed objects are, among others, print temperature, degree of filling, layer thickness, and surface development.
- the PLA polymer can be used in implants as a carrier of medical substances released only after implantation in the patient’s body.
- computer planning can be the basis for considering and planning the production process, which translates into the optimum use of material and energy for production.
- 3D printing reduces waste and can revolutionize logistics and reduce costs and distribution time.

The article shows the possibility of modifying PLA properties through layered printing or modifying PLA filaments.

Additive manufacturing should aim at working in a closed cycle and focus on biodegradable raw materials. To achieve this, it is necessary to study biodegradable polymers’ connections, modifications, and functionalization. This approach allows for producing objects with the expected strength properties from eco-friendly products. The interest in multi-material printing is growing. By analyzing these trends in the long-term perspective, the optimal parameters will be better known, and the recycling of materials will be more efficient.

## 8. Limitations

There are limitations to this study: printing on multiple materials is a relatively new trend in additive manufacturing. The authors test different samples—the print tests are not standardized. Standards for polymers are the most frequently used. It is impossible to compare tests on samples produced additively and unambiguously.

## Figures and Tables

**Table 1:** Summary of information on layered printing for selected materials.

Material	Sample Geometry	Research	Variable Parameters	Best Results
PLA/TPU [84]	Dog bone sample	Ultimate stress: 64–68 MPa Young’s Modulus 1040–1075 MPa	Mechanical interlocking systems: T-shape, U-shape, and dovetail shape	T-shape is the best locking mechanism for the TPU-PLA combination
PLA/TPU [77]	Cylindrical	Shear strength 0.63 MPa	Order of printing layers Surface pattern (linear, concentric)	TPU-linear pattern 0° and PLA-linear pattern 45°
PLA-TPU, CPE-PLA [85]	Rectangular prism	Elongation—the force of adhesion Peak stress from 0.28 MPa to 1.32 MPa	Mechanical Interlocking Order of printing layers Surface expansion	PLA-TPU + Mechanical Interlocking
PLA, PLA/PVC, PLA/wood powder, PLA/magnetite (Fe_3_O_4_) [86]	Dog bone sample	Tensile strength 41.65 MPa	Infill density Infill angle Infill speed	Infill density 100%, Infill angle of 45° and Infill speed of 90 mm/s
ABS, CF-PLA [87]	Square laminar sheets	Uniaxial tensile load Bond strength 45 MPa	Printing speed Infill density Layer height Layer thickness Ratio	The printing speed of 50.54 mm/s, Infill density of 79.82%, Layer height of 0.15, the Layer thickness ratio of 0.49
ABS, CF-PLA [90,91]	Impact testing sample Dog bone sample	Impact strength from 7672.9 to 23,465.6 kJ/m^2^ Elastic modulus = 2204.45 MPa; Ultimate strength = 51.34 MPa Elongation = 9%	Using external walls in mesh structures Using ABS for strengthening CF-PLA	Higher impact strength (280 to 365%) compared to CF-PLA samples Printing parameters: speed: 20 mm/s, infill density: 67.838%, layer height: 0.23 mm and clad ratio: 0.25
ABS, PLA, HIPS [93]	Dog bone sample	Tensile strength: 44.4 MPa Young’s modulus 1364.25 MPa	Order of printing layers from different materials	Best configuration PLA -ABS-PLA
PLA+ PA6-TiO_2_ [97]	Dog bone sample	Strength 61 MPa	Printing speed Layer combinations Infill pattern	Printing speeds 90 mm/s rectilinear fill pattern 5 PLA layers and five composite layers were the best combinations
PLA/PBAT/PBS [100]	Dog bone sample	Tensile strength 50.4 MPa Young’s Modulus 1 GPa	Different material proportions in the composite	The best roughness and dimensional accuracy parameters were obtained for the proportion 70/10/20/10 The addition of PBS and nano talc increased the PLA crystallinity: Storage modulus, Tensile and flexural Strength Anisotropic characteristics

**Table 2:** Summary of information on selected modified filaments.

Material	Methodology	Result
PLA with the addition of almond peel powder [101]	Shear resistance using cancellous screw	Shear strength at peak (23.02 MPa) Maximum shear strength at break (22.90 MPa) for the honeycomb infill pattern at 100% screw insertion and 30° rake angle
PLA with polypropylene [102]	Overwhelmed physical interlocking and minimum chemical grafting	High structural stability (mechanical and intermolecular) to thermal degradation, compared to pure PLA
PLA with silicon nanocomposite (clay nanocomposite) [103]	Changing the printing temperature, verifying sample transparency	Increase in thermal stability and modulus of elasticity The samples become more transparent as the printing temperature increases
PLA with silica (silica-silicon dioxide SiO_2_) [104]	Addition of 10% of silica by weight	Increase in tensile strength up to 121 MPa
PLA with flax fibers [105]	Adding flax, testing the porosity of the fiber	Material gaps and weakening of material bonds
PLA with wood [107]	Examination of microstructure and mechanical properties Shape changes under the influence of climatic conditions [106]	Optimum printing temperature—220 °C The higher the wood content, the greater the observed shape change
PLA with mango extract [109]	Examination of bioactive properties	3D printing polymers can be made bioactive directly using natural extracts
PLA with methotrexate [110]	Examination of the release time of the drug	Print releases the active substance at the site of implantation for more than 30 days
PLA with PCL polycaprolactone and HA hydroxyapatite [111]	Strength test depending on the hydroxyapatite content Assessment of cytotoxicity and biocompatibility	Compressive strength 82.72 MPa, tensile strength 52.05 MPa with a hydroxyapatite content of 15% Cells are viable and can increase on frames, the most favorable weight ratio of PLA/PCL—70/30
PLA with TPU and an antibiotic [115]	Mechanical, structural, microscopic, and degradation analysis	TPU/PLA ratio—12:1 The antibiotic—amikacin is stable during extrusion at elevated temperatures
PLA with TPU [116]	Sterilization test	Possibility of using PLA with TPU for personal protective equipment—the ability to re-sterilize
PLA with TPU and graphene oxide GO [118]	Influence of graphene oxide on mechanical and biocompatible properties of prints	The addition of GO improves the mechanical properties by 167% for the compression modulus and 75.5% for the tensile modulus PLA/GO frames are biocompatible; they promote cell proliferation and mineralization

## Data Availability

Not applicable.
